# Draft Genome Sequence of Rhizobium tropici SARCC-755, a Free-Living Rhizobium That Nodulated and Promoted Growth in Pigeonpea [Cajanus cajan (L.) Millsp.]

**DOI:** 10.1128/MRA.01122-19

**Published:** 2020-01-09

**Authors:** Francina Lebogang Bopape, Ahmed Idris Hassen, Zacharias H. Swanevelder, Eastonce T. Gwata

**Affiliations:** a Agricultural Research Council, Plant Health and Protection (ARC-PHP), Pretoria, South Africa; b Agricultural Research Council, Biotechnology Platform (ARC-BTP), Onderstepoort, Pretoria, South Africa; c Department of Plant Production, School of Agriculture, University of Venda, Thohoyandou, South Africa; University of Maryland School of Medicine

## Abstract

Rhizobium tropici SARCC-755 is a free-living soil bacterium that formed nodules on pigeonpea roots in the present study. However, the draft genome sequence reveals that this *Rhizobium* species contains the *nolR* gene but lacks the common nodulation (*nodABC*) genes and probably uses other pathways to induce nodules on the legume plant.

## ANNOUNCEMENT

**FIG 1:**
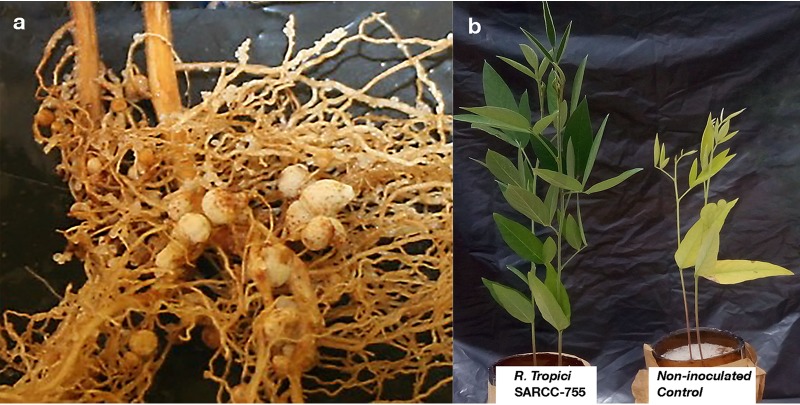
(a) Inoculation of Cajanus cajan with Rhizobium tropici SARCC-755 in a glasshouse experiment resulted in the formation of big pink root nodules. (b) Pigeonpea plants inoculated with the rhizobium look healthy and green (left) compared with the noninoculated plants with stunted growth and yellow leaves (right).

Rhizobial strains that showed low levels of DNA-DNA hybridization with Rhizobium leguminosarum and genetic distances well beyond the acceptable threshold that separates bacterial species have been reclassified as Rhizobium tropici (1). Here, we report the draft genome sequence of a free-living Rhizobium tropici strain, SARCC-755, that initially survived in local virgin soil without any symbiotic association with legumes. This strain was trapped by the pigeonpea root nodules using a soil trap experiment in a glasshouse, after which morphologically different strains of rhizobia were isolated from the nodules using standard microbiological procedures (2, 3). The isolates were subjected to a gnotobiotic nodulation experiment on the same legume using the Leonard jar assembly method (3). One of the strains, designated Rhizobium tropici SARCC-755 after a 99.9% 16S rRNA sequence similarity with *R. tropici* was determined, resulted in the formation of several big nodules on the legume Cajanus cajan with significant growth promotion (Fig. 1). This draft genome sequence was generated to better understand the symbiotic properties of this bacterium and to elucidate the genetic determinants responsible for nodulation and nitrogen fixation in this legume.

For DNA extraction, a single pure colony of the bacterium grown on yeast mannitol (YM) agar was transferred to tryptone yeast (TY) broth medium and incubated for 3 days on a rotary shaker (150 rpm) at 28°C. A total of 1.5 ml of the broth culture was used to extract DNA using the Wizard genomic DNA extraction kit according to the manufacturer’s instructions (Promega, Madison, WI, USA). DNA libraries were prepared using the Nextera protocol (Illumina, USA) and paired-end (300 × 2 bp) sequenced on a MiSeq (Illumina) sequencer at the Biotechnology Platform, Agricultural Research Council (Onderstepoort, South Africa). A total of 10,364,436 raw sequence reads were obtained. Adapter sequences and low-quality bases were trimmed, and the overlapping pairs were merged using CLC Genomic Workbench version 8.5.1. A total of 8,265,062 reads were thus obtained and used in a *de novo* assembly that resulted in a draft genome sequence of 6,300,547 bp consisting of 36 contigs (including scaffolds) with a GC content of 59.9% and an *N*_50_ value of 600,084 bp. The maximum scaffold size was 876,087 bp. Gene annotations were performed with the NCBI Prokaryotic Genome Annotation Pipeline (PAGP) (4) and resulted in a total of 6,309 coding regions, 45 tRNAs, and 3 rRNAs dispersed in the genome.

This rhizobium shows the ability to form nodules on the roots of pigeonpea, but except for the *nolR* gene that acts as the DNA binding transcription factor, it lacks the common nodulation (*nodABC*) genes. This was confirmed by mapping the reads to the full genomes of *R*. *tropici* CIAT 899 and other existing islands, where none of the reads map to the *nod* and *nif* genes of the reference genomes. It is therefore possible that this free-living *R. tropici* strain uses purine biosynthesis precursors (5) such as inosine-5-monophosphate, AMP, and adenylosuccinate, all detected in this draft genome sequence, as alternative pathways for inducing nodulation, which warrants further study.

### Data availability.

This whole-genome shotgun project has been deposited at DDBJ/ENA/GenBank under the accession number VNIP00000000, and the version described in this paper is version VNIP01000000. The SRA accession number for the raw data is PRJNA556568.

## References

[1] Martínez-Romero E, Segovia L, Mercante FM, Franco AA, Graham P, Pardo MA 1991 *Rhizobium tropici*, a novel species nodulating *Phaseolus vulgaris* L. beans and *Leucaena* sp. trees. Int J Syst Bacteriol 41:417–426. doi:10.1099/00207713-41-3-417. 1715738

[2] Odee DW, Sutherland JM, Makatiani ET, McInroy SG, Sprent JI 1997 Phenotypic characteristics and composition of rhizobia associated with woody legumes growing in diverse Kenyan conditions. Plant Soil 188:65–75. doi:10.1023/A:1004204413140.

[3] Hassen AI, Bopape FL, Lamprecht SC, Habig J 2012 Nodulation of rooibos (*Aspalathus linearis* burm. f.), an indigenous South African legume, by members of both the α-Proteobacteria and β-Proteobacteria. Biol Fertil Soils 48:295–303. doi:10.1007/s00374-011-0628-3.

[4] Tatusova T, DiCuccio M, Badretdin A, Chetvernin V, Nawrocki EP, Zaslavsky L, Lomsadze A, Pruitt KD, Borodovsky M, Ostell J 2016 NCBI Prokaryotic Genome Annotation Pipeline. Nucleic Acids Res 44:6614–6624. doi:10.1093/nar/gkw569. 27342282 PMC5001611

[5] Giraud E, Moulin M, Vallene D, Barbe V, Cytryn E, Avarre J-C, Jaubert M, Simon D, Cartieux F, Prin Y, Bena G, Hannibal L, Fardoux J, Kojadinovich M, Vuillet L, Lajus A, Cruveiller S, Rouy Z, Mangenot S, Segurens B, Dossat C, Frank LW, Chang W-S, Saunders E, Bruce D, Richardson P, Normand P, Dreyfus B, Pignol D, Stacy G, Emerich D, Vermegilo A, Medigue C, Sadowsky M 2007 Legumes symbioses: absence of nod genes in photosynthetic *Bradyrhizobia*. Science 326:1307–1312. doi:10.1126/science.1139548. 17540897

